# Taking patient reported outcomes centre stage in cancer research – why has it taken so long?

**DOI:** 10.1186/s40900-018-0109-z

**Published:** 2018-07-19

**Authors:** Peter Selby, Galina Velikova

**Affiliations:** Leeds Institute of Cancer and Pathology, University of Leeds and Leeds Cancer Centre, St James’s University Hospital, Beckett Street, Leeds, LS9 7TF England

## Abstract

**Plain English summary:**

Roger Wilson challenged cancer professionals in research and care to place the patient perspective and patient reported outcome measures centre stage. The ability to collect information from patients using structured questionnaires (known as Patient Reported Outcome Measures or PROMs) which ask about clinical issues (such as disease symptoms or treatment side-effects) as well as social, emotional and psychological issues has existed for 40 years. They provide a powerful way of working out whether an aspect of diagnosis or treatment for cancer is bringing real benefits to patients that can be measured using these structured questionnaires. When they are used, studies and cancer service developments are clearly constrained to focus on what matters to patients rather than, perhaps what matters to health service professionals or recent exciting but perhaps relatively unproven new technologies. There is good evidence that PROMs can contribute valuable inputs into the results of randomised controlled trials, clinical consultations and surveys of whole populations even at a national level. However, there is a great deal more work to be done on methodology and perhaps to change attitudes and cultures within the healthcare professions before they can deliver all of their potential to bring benefits to cancer patients.

**Abstract:**

In response to Roger Wilson’s challenge to place a patient-centred approach using Patient Reported Outcome Measures (PROMs) across all of the patient pathway, we have summarised progress over 40 years. We have critically evaluated what has been achieved to use patient reported outcomes in randomised controlled trials, in routine clinical practice and in population surveys. We conclude that there has been substantial scientific progress but that it has, arguably, been relatively slow. Barriers to placing PROMs centre stage in all of these areas of activity remain in methodology and to a degree in professional attitudes and culture. Active research programmes on methodology and closer working between healthcare professionals, cancer patients and patient advocates are the key requirements to speed up the use and application of PROMs and which should bring benefits to cancer patients and healthcare services.

We write in response to Roger Wilson’s thoughtful, stimulating and challenging paper “Patient led PROMs must take centre stage in cancer research”, published in Research Involvement and Engagement. In the article he takes an overview of cancer research, recognising the progress that has been made both in cancer diagnosis and treatment and in patient outcomes, but enters substantial and important challenges. Clinical researchers and health service developers have yet to place the patient perspective and the patient-centred approach centre stage. We have failed to exploit the opportunities presented by Patient Reported Outcome Measures (PROMs) that can help us do so. We essentially agree with Roger Wilson. We have to do much better in placing patient perspective at the centre of cancer research and of cancer care. We also agree that in doing so we will take important steps towards tackling some of the practical and cultural challenges which we have failed to address adequately. The clinical and research community remain much too ready to prioritise and emphasise exciting new technologies whilst neglecting relatively simple but firmly evidence based interventions which have the potential to greatly improve patient outcomes not only in terms of their survival but also in terms of better patient experience and quality of life.

We are all rightly excited by the promise of new technologies in immunotherapy, genetics and personalised medicine. However, as Roger Wilson points out, they remain a long way from delivering global improvements in cancer outcomes. On the other hand simple measures around public awareness, access to therapy, ensuring that well established best practice is used widely across healthcare systems and across different countries, and the appropriate delivery of timely diagnosis and multidisciplinary specialised care have proven potential to improve outcomes. It has been recently suggested [1] that if we made proper use of well established existing good practice, we could continue to improve the survival of cancer patients from the approximately 50% long term survival achieved in many European countries towards the 60% long term survival that is achieved through best practice in some European countries. It has also been estimated that if we make well planned and effective use of research in new diagnostic and treatment approaches derived from existing technologies, then it is realistic to aim for a 70% long term survival or even better in as little as two decades. However, if we are to achieve this we have to understand the issues that Roger Wilson emphasises. We must ensure that we make the best use of what is known whilst we also quickly take advantage of exciting new cancer sciences.

The use of PROMs to evaluate progress will mean that care and research are focused to address the issues that really matter to patients. The wide uptake of Patient Reported Outcomes and the impact that this has on the culture of healthcare professionals will ensure that services as a whole deliver what patients expect.

Unfortunately, the time lag between work on Patient Reported Outcome Measures and their comprehensive position in centre stage in cancer research is uncomfortably long. PROMs in the form of health related quality of life questionnaires were first proposed in 1976 by Terry Priestman and Michael Baum in a seminal article in the Lancet [2]. Their contribution was followed reasonably quickly by programmes of work which took the idea of health related quality of life and applied the concepts of psychometrics to the development of the relevant questionnaires, the evaluation of their psychometric properties and their performance in the evaluation of innovations [2–5]. So by the early 90s we had well evaluated formal tools that could be deployed in cancer research and in cancer care with some confidence in their performance. At that point workers in the field faced many challenges. We can characterise three key questions:

## Can PROMs be used in randomised prospective trials to collect data in a way that allows a statement to be made about the impact on patient’s quality of life including not only the activity of the disease, but also emotional, social, physical wellbeing?

Broadly the answer to the question about the value of PROMs in clinical trials is strongly positive [6]. Building particularly on the work of the EORTC (European Organisation for Research and Treatment of Cancer) with their core Quality of Life questionnaire, QLQ-C30, and David Cella and colleagues in North America with the FACT questionnaires, it is possible to have comparative data in a clinical trial that can give powerful relative statements about how one group of patients compare to another. It is still difficult to make comparisons between different questionnaires in different studies but the relative performance of treatment strategies in answering the specific questions that are posed within the trial has been valuable. A powerful recent example is the Prostate Testing for Cancer and Treatment (ProtecT) trial addressing very important and challenging questions around the initial management of men with prostate cancer. The rigorous and extensive use of PROMs in that study allow us to see that even though there are important specific damaging effects of different treatments which may have important consequences for sexual and urological outcomes for men, overall the quality of life achieved is eventually remarkably good for each of the different treatment approaches [7]. Systematic reviews have looked at the value of PROMs in aiding decision-taking based upon randomised controlled trials. It is possible to include that the PROMs do aid decision-making in a range of clinical trial settings [8]*.*

## Is it possible to use PROMs in routine clinical care to improve consultations and clinical outcomes?

In some ways the challenge of using PROMs in routine clinical care is methodologically even greater than their use in randomised trials. In clinical care the information has to be meaningful to both patient and clinician and be accessible and useable in the consultation in real time. Absolute interpretations are much more important in this setting because there are no randomised controlled groups for comparison.

This question has been studied carefully by a large number of groups over the last two decades [9]. Jensen et al. [9] and Kotronoulas et al. [10] have completed systematic reviews quite recently of the 25 randomised controlled trials of this approach. They concluded that there was still significant gaps in the evidence base but there is indeed growing evidence in support of the use of routine PROM collection and the demonstration that it can enable better patient-centred care in the setting of cancer. However*,* it is worth noting the results of a recent randomised trial of *systematic monitoring of* patients’ symptoms using electronic PROMs which showed improved clinician awareness of symptoms, better symptom management, less emergency visits, better quality of life and improved overall survival in patients receiving chemotherapy for advanced cancer [11, 12].

## Can we use PROMs in a meaningful way to survey whole populations of cancer patients and the general population?

It is now almost a decade since the Darzi Report recommended that PROMs data should be an essential component of healthcare evaluation in the United Kingdom and the commitment was made in the NHS to use PROMs in the development of world-class cancer outcomes [13, 14]. There is progress. Large scale surveys of prostate cancer survivors [15] and colorectal cancer patients [16] are generating valuable information on patient reported outcomes in national populations. They demonstrate broadly that it is possible to achieve comparable quality of life of the cancer patient after cancer treatment to the quality of life of the general population although often with specific deficits that require attention. The commitment to move PROMs centre stage in the UK NHS as part of the Cancer Dashboard is to be welcomed but also approached cautiously because there are still methodological challenges. Five pilot sites across England are currently exploring ways to collect routinely PROMs in the NHS to inform a national roll-out.

So we can point to quite hard evidence of reasonable quality and quantity which shows us that PROMs can contribute in randomised trials, routine practice and in population surveys. So why has it taken 40 years to reach this point and why, as Roger Wilson correctly points out, are PROMs not centre stage in either cancer research or cancer care? So some possible explanations are worth consideration:

### Psychometrics is a difficult science

There has to be a rigorous and relevant evaluation of a performance of the questionnaires not only to ensure that they are reliable and valid but also to be clear that they tap into the appropriate domain of patient experience which they are seeking to measure. Patient input here is essential and is a longstanding requirement of the evaluation of psychometric properties of questionnaires. However, the process is time consuming, sometimes quite expensive and can involve large numbers of patients or the general population to achieve meaningful results. Individuals and groups have tended towards “ownership” of their questionnaires. This may well reflect the efforts required to evaluate them adequately, but has led to a lack of general consensus in the field and a confusing number of questionnaires. This has slowed down the generalisation and accessibility of these methods in cancer research and care.

### There are still pockets of professional resistance to the engagement and empowerment of patients and to efforts to tap into patient’s perceptions through the use of PROMs

This remains the case despite the existence of evidence that an engaged and empowered patient population can promote better and more cost effective healthcare delivery [17].

### There are external pressures on cancer professionals and cancer patients which shift the focus away from the patient perception and patient-centred care

Cancer sciences are truly exciting currently and funding bodies, individuals and research institutions are all seeking to demonstrate their success and prowess in such exciting modern science. Indeed the psychosocial cancer research community was horrified some years ago when one of its most major and respected funders decided that research in psychosocial oncology was no longer a strategic priority for the organisation, and withdrew their funding in this area in order to focus on research that would lead towards cancer cure. This view fails to take on board the importance of establishing a strong patient perspective to ensure that those responsible for cancer services remain grounded in the deliverable evidence that is improving long term survival, as was discussed at the beginning of this article.

### The Way Forward

So if we take on board Roger Wilson’s analysis and challenges what are the practical steps that may move PROMs centre stage in cancer research with the attendant benefits to cancer services, cancer patients and cancer outcomes?

- We do need to continue and sustain our efforts on methodology. As Roger Wilson points out, integrating quality of life measures across the whole patient pathway and not, as most commonly in randomised trials, to a single post-treatment timepoint. Quick and easy, reliable and valid questionnaires continue to elude us in many settings despite the importance that is attached to quality adjustment of survival by many healthcare systems. The increased accessibility of electronic data collection via Internet and mobile devices together with the wide use of Electronic Patient Records featuring patient portals makes this approach possible and feasible.
- The voice of cancer patients and patient advocates is increasingly powerful with cancer professionals, health service leaders and managers and government health departments across the globe. It is being and can be further raised to point out the value of Patient Reported Outcome Measures in ensuring that research and care and service development are firmly grounded in the things that matter to patients as a whole and things that are likely to work. We need to redouble our efforts to simplify and clarify and make more coherent messages for patients and professionals that will keep them focused on improving outcomes that matter to patients.
- Patient and public involvement as research partners should be increasingly a core feature in PROM development and application. Such involvement may further contribute to the development of PROMs that really do measure what matters, and are relevant and acceptable to the end users.
- There are important training and education challenges in all healthcare professionals and oncology disciplines about patient-centred care and the value of PROMs as a tool to support communication and shared decision-making to ensure effective patient-centred care [18].
- The dialogue about strategy, cancer research and cancer care has to become a more evenly balanced dialogue between patients, professionals, healthcare commissioners and politicians.

Debates on cancer policy are inevitably going to become more complex. However, a balanced approach that involves professionals, patients and is informed by Patient Reported Outcome Measures is much more likely to generate progress in cancer research and cancer care development that improves outcomes, patient experience and quality of life [1].
